# Prevalence and Time Trends of Low Serum B12 Levels and Inadequate B12 Dietary Intake in Lebanese Adults amidst the Food Insecurity Situation: Findings from a Nationally Representative Cross-Sectional Study

**DOI:** 10.3390/nu16020226

**Published:** 2024-01-10

**Authors:** Maha Hoteit, Razan Khadra, Zahraa Fadlallah, Youmna Mourad, Mohamad Chahine, Farouk Skaiki, Elham Al Manasfi, Abdulrahman Chahine, Omasyarifa Binti Jamal Poh, Nikolaos Tzenios

**Affiliations:** 1 Food Sciences Unit, National Council for Scientific Research-Lebanon (CNRS-L), Beirut P.O. Box 11-8281, Lebanonzh.fadlallah@gmail.com (Z.F.); 2 Faculty of Public Health, Section 1, Lebanese University, Beirut P.O. Box 6573, Lebanon; 3 Al Hadi Laboratory and IVF Center, Beirut 1103, Lebanon; youmna_mourad@hotmail.com; 4 Biological and Chemical Technology, Kursk State Medical University, 305000 Kursk, Russia; chahine@kgmu.com; 5 Department of Molecular Biology, General Management, Al Karim Medical Laboratories, Saida 1600, Lebanon; alkarimlaboratory@hotmail.com; 6 Arab Group for Scientific Research, Beirut 1103, Lebanon; ilhammanasfi@gmail.com; 7 Radiology Department, Beirut Arab University, Beirut 1107, Lebanon; abderahmans@yahoo.com; 8 Obstetrics and Gynecology ICE, Kursk State Medical University, 305000 Kursk, Russia; drmno131@yahoo.com; 9 Faculty of Public Health, Charisma University, London EC1V 7QE, UK

**Keywords:** vitamin B12, food insecurity, intake, serum level, Lebanon

## Abstract

Rising food insecurity (FI) and the increased prices of animal-based foods could compromise vitamin B12 (B12) intake and serum levels in food-insecure people. Our study aims to determine the prevalence of low levels of serum B12 and its inadequate dietary intake among a nationally representative sample of Lebanese adults aged 18 to 64 years, while exploring the impact of FI and identifying other potential predictors. The B12 intake was assessed using a food frequency questionnaire and the mean of two non-consecutive 24 h recalls. The B12 serum levels were also examined. To examine the time trends in the B12 serum levels and dietary intake, the data from a large representative digitized database of 6290 participants were collected, along with the examination of FAOSTAT food consumption data both before and during the economic crisis period. Our findings revealed that 47.3% of households in Lebanon experienced FI. The inadequate intake of B12 food sources was prevalent in 52.5% of participants, and 61.1% presented with low (23%) or intermediate (38.1%) levels of serum B12. The food-secure households had a higher proportion of B12 intake from meats (*p* = 0.004), while traditional foods contributed more to food-insecure households (*p* = 0.000). The participants who were female, unemployed, food-insecure, and had low dietary diversity were about two to three times more likely to have inadequate B12 intake. The predictors of low levels of serum B12 included residing in Beqaa (OR = 2.856 and 95% CI = 1.018–8.01) and having inadequate B12 intake (OR = 1.788 and 95% CI = 1.2–2.65). The findings from observing the time trends in the consumption and the serum levels of B12 indicate a considerable decline in the consumption of most animal-based foods compared to the pre-crisis period, associated with a decline in the serum levels of B12. In conclusion, an alarming prevalence of low dietary intake and low serum levels of B12 was revealed among Lebanese adults, and the indirect effect of FI on B12 serum levels mediated through B12 intake was inferred.

## 1. Introduction

Food insecurity (FI) is a widespread challenge with diverse causes, including economic, political, social, environmental, and systemic factors [1,2]. 

The state of food insecurity is of particular concern in Lebanon, since the country is facing its own unique set of adversities. The economic crisis that began in 2019 has had a profound impact on Lebanon, resulting in a severe and ongoing devaluation of the Lebanese pound, a surge in inflation, and the lifting of subsidies [3]. These developments, compounded by the impact of the COVID-19 pandemic and the 2020 Beirut port explosion, have had a detrimental effect on the food security situation in Lebanon, exacerbating the already-challenging global context. According to the World Food Programme (WFP) survey estimates, moderate or severe FI in Lebanon saw a 23-percentage point increase from April/May 2020 (21.2%) [4], near the beginning of the crisis, to November/December 2021 (44%) [4,5]. The prices of animal-based foods, such as meat, dairy products, fish, and eggs, experienced substantial increases ranging from 2225% to 3262% between October 2019 and December 2022, according to WFP estimates [5]. Recent research conducted between June and July 2022 also revealed that a large percentage (80%) of the Lebanese population consumed these food items for three days or fewer per week [6,7]. Since foods of animal origin are the primary source of vitamin B12 (B12) in the diet, the combination of rising prices and the limited consumption of these foods within the context of increasing FI in Lebanon raises concerns about the adequacy of the intake of B12 and its associated serum levels in the Lebanese population.

Low levels of serum B12 are associated with a range of adverse symptoms which can be mild to severe, including megaloblastic anemia, neurological damage, cognitive impairment, cardiovascular effects, etc. [8]. A clinically low level of B12 with classic hematological and neurological symptoms is relatively uncommon, with serum B12 levels < 200 pg/mL typically used as the defining criterion. On the other hand, a subclinical deficiency, which is associated with low or borderline B12 levels but usually no symptoms of deficiency, affects a more significant portion of the population [9]. 

The main causes of low levels of serum B12 are malabsorption; medications such as proton pump inhibitors (PPIs), histamine H2 receptor antagonists [10], metformin [11], antiepileptic drugs [12], and colchicine [13]; and inadequate intake. Moreover, various conditions can impair the absorption of B12, including pernicious anemia and gastric bypass surgery [14]. Atrophic gastritis [14], a condition that is common in the elderly [15], and damage to the terminal ileum caused by surgical removal or inflammatory conditions such as Crohn’s disease or celiac disease can also impair the absorption of B12 [14]. As for the dietary causes of deficiency, prolonged insufficient intake of B12 may deplete the liver’s stores of the vitamin, resulting in its deficiency. This phenomenon is evident in individuals who follow a vegan diet and consume a limited amount of B12 [16]. While it may take a relatively long time for the B12 stores to be depleted, once depleted, the symptoms of deficiency may occur rapidly, of which some are irreversible [14]. 

Although there is considerable evidence about the impact of FI on the intake and serum levels of certain micronutrients, research specifically examining the relationship between FI and vitamin B12 intake or serum levels, especially in adults, is scarce. For instance, among the limited studies available, data from the United States (US) based on the 2001 NHANES surveys showed that the prevalence of low dietary intake of B12 was higher in adults from food-insecure families (13.4%) compared to those from food-secure families (9.7%) [16]; however, this difference was not a significant one. Likewise, there was no significant difference in the mean serum levels of B12 (pg/mL) between adults from food-insecure (511.4) and food-secure families (477.2), with a *p*-value of 0.84 [16]. In contrast, a separate study conducted in rural America found a significant difference in the prevalence of adequate B12 intake between food-insecure (64.3%) and food-secure adults (71.9%) [17]. Despite these findings, there remains a lack of comprehensive research in the literature on the impact of FI on B12 intake and status.

In Lebanon, FI was shown to affect food consumption patterns [6,7]; however, to our knowledge, no studies have been conducted in the country exploring the effect of FI on B12 intake or serum levels, which adds to the need for our study as the economic situation continues to deteriorate and as the prevalence of FI is on the rise. Accordingly, our study aims to determine the prevalence of inadequate intake of B12 food sources and the low levels of serum B12 among a nationally representative sample of Lebanese adults aged 18 to 64 y, while exploring the impact of FI and identifying other potential predictors.

## 2. Materials and Methods

### 2.1. Study Design and Eligibility Criteria

This is a cross-sectional study which was conducted over a period of 5 months between May and September 2022. The eligibility criteria were carefully chosen to ensure that the study’s objectives were met and to provide a representative sample of the study population. The inclusion criteria were adults aged 18 to 64 years of Lebanese nationality. The exclusion criteria included lactating and pregnant women; strict vegetarians or vegans; alcoholics; those having gastrointestinal disorders (inflammatory bowel disease or Celiac’s disease), chronic diarrhea, Helicobacter pylori infection, pernicious anemia, tuberculosis, or malignancy; those taking vitamin B12 or multivitamins containing vitamin B12 successively for the 6 months prior to participation in the study; those taking medications with known effects on serum B12 concentration (e.g., acid-lowering medications, metformin, or medications containing metformin, corticosteroids, colchicine, and anti-epileptic drugs) for a successive period of 3 months before enrollment; and those having previously undergone gastrointestinal surgeries (e.g., gastrectomy, gastric bypass, or removal of the ileum).

### 2.2. Sampling Method and Recruitment Process

An estimated minimum sample of 400 participants was required for national representativeness, calculated based on the population estimates from 2018 to 2019 using the formula (n = [p (1 − p)] × [(Z_∝/2_)^2^/(e)^2^), where n denotes the sample size; Z_(∝/2) is the reliability coefficient of the standard error at a 5% level of significance = 1.96; p represents the probability of adults (18–64 y) who were unable to practice preventive measures of the diseases (50%); and e refers to the level of standard error tolerated (5%), as stated by Hosmer and Lemeshow [18]. In our study, 449 participants (265 females and 184 males) were recruited from all 8 governorates across Lebanon using a combination of stratified cluster sampling, with the stratified groups being male and female and the clusters being the governorates. 

The recruitment process initially involved multiple channels, including individual volunteers, first aid centers, charitable organizations, and medical centers, to reach a broad range of participants within their outreach. The participants who were recruited through these channels were encouraged to refer other individuals within their networks. This sampling approach enabled access to populations that were physically difficult to reach due to time and budget constraints. The individuals who showed interest in participating were informed about the nature of the study and provided implied verbal consent, after which they were screened for eligibility. The eligible participants were then asked to provide electronic written consent before proceeding with the study phases. To ensure a broad representation of Lebanese households in the study, a maximum of one individual per household was allowed to participate.

### 2.3. Data Collection

#### 2.3.1. Phase 1: Administration of Sociodemographic Questionnaire 

The first phase involved collecting data via a pre-tested questionnaire that was completed during an interview with the participants and consisted of 3 sections. The first section included questions regarding demographic and socioeconomic information, as well as medical history. The questions about the number of persons and rooms in the household were included in order to calculate the crowding index (CI), which is a proxy indicator of household socioeconomic status [19,20]. The second section included the Arab Family Food Security Scale (AFFSS) of 7 items, already validated in the Lebanese population, where participants were asked a series of questions related to their household’s food security [21]. In the third section, the Food Consumption Score (FCS) was used to assess the DD of households, which is the number of individual foods consumed over a reference period. The FCS is calculated by determining the frequency of the consumption of different food groups by a household during the 7 days preceding the survey [22]. It also included questions about the number of meals consumed by the person or their household the day before and if this reported intake was as usual.

#### 2.3.2. Phase 2: Food Frequency Questionnaire and 24 h Recall 

After completing the sociodemographic questionnaire, a 157-item semi-quantitative food frequency questionnaire (FFQ), previously validated among the general adult Lebanese population [23], was administered by trained dietitians in 30 min phone interviews. The FFQ included questions about the frequency of the consumption of various food items in the past 6 months. Moreover, in an effort to investigate the dietary intake of the study population, two non-consecutive dietary recalls were administered across weekdays and weekends. Both the FFQ and the two 24 h recalls were conducted during the same time period. The participants were provided with instructions and visual aids to help them to recall their food consumption and to estimate portion sizes as accurately as possible. During the interview, the responses were recorded by the interviewers as the number of servings consumed and the frequency of the consumption of the reported servings (daily, weekly, or monthly) for each food item. 

#### 2.3.3. Phase 3: Blood Sampling and Anthropometrics

In the third phase of the study, blood samples and weight and height measurements were taken. The venous blood samples were collected by trained laboratory technicians in clot activator tubes containing gel for B12 tests and ethylenediaminetetraacetic acid tubes (EDTA) for CBC complete blood count (CBC) tests. 

The serum vitamin B12 analysis was performed using a Roche Cobas immunoanalyzer (Roche Diagnosis, Indianapolis, IN, USA) [24]. The principle of the method is an electrochemiluminescence immunoassay (ECLIA) using intrinsic factors specific for vitamin B12. Vitamin B12 in the sample competes with the added vitamin B12 labeled with biotin for the binding sites on the ruthenium-labeled intrinsic factor complex.

The results were determined via a calibration curve, using an instrument specifically generated using two different standards. As for the performance characteristics of the assay, the analytical sensitivity was 30 pg/mL or 22.0 pmol/L; the linearity was 50–2000 pg/mL; the precision% was 2.4–5%; and the reference range (2.5th–97.5th percentile) was 197–771 pg/mL [24].

**Figure 1:**
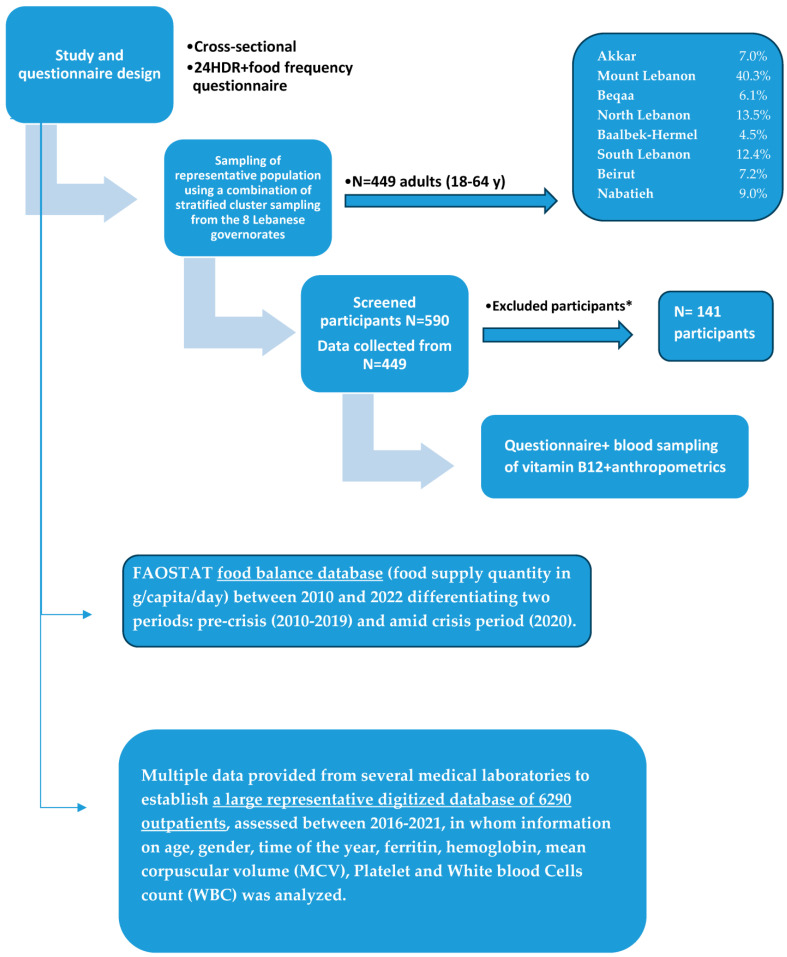
Flow chart of the recruitment process and data collection in the study. * Exclusion criteria: lactating and pregnant women; strict vegetarians or vegans; alcoholics; those having gastrointestinal disorders (inflammatory bowel disease or Celiac’s disease), chronic diarrhea, Helicobacter pylori infection, pernicious anemia, tuberculosis, or malignancy; those taking vitamin B12 or multivitamins containing vitamin B12 successively for the 6 months prior to participation in the study; those taking medications with known effects on serum B12 concentration (e.g., acid-lowering medications, metformin, or medications containing metformin, corticosteroids, colchicine, and anti-epileptic drugs) for a successive period of 3 months before enrollment; and those having previously undergone gastrointestinal surgeries (e.g., gastrectomy, gastric bypass, or removal of the ileum).

The weight and height measurements were taken using standardized protocols and calibrated equipment, i.e., a digital scale for weighing and a stadiometer for measuring heights. Both the weight and height were measured three times for each participant for accuracy, and the average of the three measurements was later calculated to compute the Body Mass Index (BMI) (Figure 1).

### 2.4. Time Trends in Consumption of Vitamin B12 Sources and Serum Levels

To investigate the time trends in the vitamin B12 consumption data and the prevalences of vitamin B12 deficiencies in Lebanon, we searched the FAOSTAT food balance database for statistics showing the food supply quantity in g/capita/day between 2010 and 2022 differentiating two periods: the pre-crisis period (2010–2019) and the period amid the crisis (2020) [25]. Moreover, we collected multiple data provided from several medical laboratories to establish a large representative digitized database of 6290 outpatients, assessed between 2016 and 2021, from whom information on age, gender, time of the year, ferritin, hemoglobin, mean corpuscular volume (MCV), platelet and white blood cells count (WBC) was analyzed. Abnormal conditions related to chronic diseases associated with abnormalities in vitamin B12 were excluded from this analysis (Figure 1). 

### 2.5. Data Management and Statistical Analysis

The data was managed and coded using Microsoft Excel 2016. The CI was computed by dividing the total number of household members (excluding infants) by the total number of rooms in the household (excluding kitchens and bathrooms). The food security status was categorized as food security (a score of 0–2), moderate FI (a score of 3–5), and severe FI (a score of 6–7) [21]. The DD was determined by calculating the FCS using the following formula:

(starches × 2) + (pulses × 3) + vegetables + fruit + (meat × 4) + (dairy products × 4) + (fats × 0.5) + (sugar × 0.5),

which was categorized as a high household DD (a FCS of ≥42) and a low household DD (a FCS of <42). The B12 intake (μg/d) for each participant was calculated from the FFQ data by adding the B12 intake from all food items. The B12 composition of most food items was determined from the USDA database [26] and was estimated for some local foods for which composition was unavailable elsewhere. The B12 intake was categorized according to the RDA cutoff as adequate (≥2.4 μg/d) and inadequate (<2.4 μg/d) [26]. As for the food group consumption (g/day) and the contributions to daily B12 intake (%), the food items were categorized into food groups using the WHO “GEMS” distribution [27]. The method used for calculating the food group consumption (g/day) was as follows.

Using the FFQ data, the daily amount consumed of each food item was calculated as follows. If a serving was reported as consumed on a weekly or monthly basis, it was divided by 7 or 30, respectively, to convert it to a daily frequency (serving/day). The resulting daily frequencies were then multiplied by the serving sizes of the respective food items (g/serving) to obtain the amount consumed of each food item (g/day).

The individual food items were then categorized into food groups using the WHO GEMS distribution. The amounts consumed for each food item were then added together to calculate the total consumption (g/day) for each food group.

The serum B12 levels were categorized as normal (>300 pg/mL or 221.4 pmol/L), intermediate (200–300 pg/mL or 147.6–221.4 pmol/L), and low serum levels (<200 pg/mL or <147.6 pmol/L) [28,29]. After checking for missing data, errors, and outliers, 5 cases were excluded from the analysis, yielding a final number of 444 participants. 

The data analysis was performed using SPSS version 25.0. For the univariate analysis, a mean ± SD (Standard Deviation) was used for continuous variables, and the frequency (N) and percentage (%) were used for categorical variables. For the bivariate analysis, a chi-squared test, student’s *t*-test, and one-way ANOVA were used to compare the differences between groups. For the chi-squared test, when the minimum expected count was less than 5, Fisher’s exact test was used instead. The multivariate analysis was performed using a binary logistic regression with the B12 status and intake as the dependent variables. Prior to performing the bivariate and multivariate analysis, the B12 status and FI status variables were dichotomized; the vitamin B12 deficiency and insufficiency were combined and referred to as a vitamin B12 deficiency; and the moderate and severe FI were combined and referred to as FI. A *p*-value of < 0.05 was considered statistically significant.

### 2.6. Ethical Considerations

This study’s protocol was reviewed and approved by the Ethical Committee at Al Zahraa University Medical Center (#57/2022) and was conducted in accordance with the ethical principles outlined in the Declaration of Helsinki. The participants gave their informed consent prior to participation and were made aware that participation was entirely optional and that they had the right to withdraw at any time. 

## 3. Results

### 3.1. Population Characteristics

The demographic and socioeconomic characteristics of the study population are listed in Table 1. Of the 444 participants included in the study, 58.8% were females, and the mean age ± SD (years) of the overall study population was 34.1 ± 12.7. Table 2 presents the population’s health characteristics. About a third of participants (33.8%) had a normal BMI and 61.9% were overweight or obese. Regarding the disease status, one-quarter of the study population reported having one or more chronic diseases.

### 3.2. Indicators of Household Food Security

Almost half (47.3%) of the households were food-insecure, including moderate (30.6%) and severe FI (16.7%), and a low DD was observed in 56.3% of households. Additionally, more than half (54.3%) of the participants reported consuming two meals or fewer the day before, and 74.3% described their reported meal consumption the day before as their usual intake (Table 3).

### 3.3. Population’s Average Food Group Consumption

The population’s dietary intake of the food groups using data from the mean of two 24 h recalls and the FFQ is shown in Table S1. 

The average consumption ± SD (g/day) of foods of animal origin were as follows: milk and dairy products (184.5 ± 164.7), meat and meat products (69.5 ± 75.1), eggs and egg products (22.2 ± 32.6), and fish and other seafood (11.4 ± 21.0). Notably, the mean intake of males was significantly greater than that of females in several, but not all, food groups, including all animal-based food groups, with *p* = 0.011, *p* = 0.000, *p* = 0.000 and *p* = 0.000, respectively (Table S1). 

### 3.4. Assessment of Vitamin B12 Intake and Status

**Figure 2:**
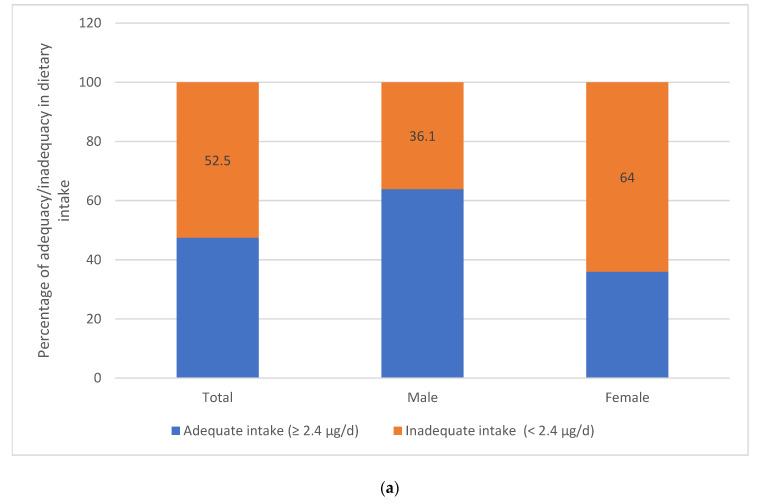

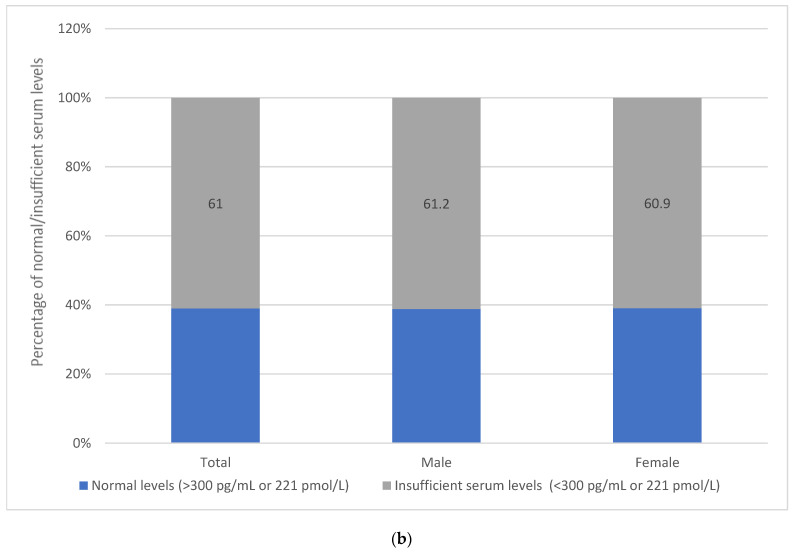
(**a**) Prevalence of inadequate vitamin B12 intake overall and by sex. B12 intake was categorized according to the RDA cutoff as adequate (≥2.4 μg/d) and inadequate (<2.4 μg/d); and (**b**) assessment of serum B12 levels overall and by sex. The serum B12 levels were categorized as normal (>300 pg/mL or 221.4 pmol/L), intermediate (200–300 pg/mL or 147.6–221.4 pmol/L), and low serum levels (<200 pg/mL or <147.6 pmol/L).

The means of the B12 intake and the serum levels of the participants were 2.9 ± 2.5 ug/d and 297.3 pg/mL, respectively. The vitamin B12 intake was higher in men (3.8 ± 3.2) compared to women (2.3 ± 1.8), *p* < 0.001. Similarly, the mean value of the B12 serum levels was 297.3 ± 158.3 with no significant difference between both genders, with *p* = 0.39. Around 61.1% of the participants presented either low levels (23%) or intermediate (38.1%) levels of B12, defined as <200 pg/mL or <147.6 pmol/L and 200–300 pg/mL or 147.6–221.4 pmol/L, respectively. Notably, 68.6% of deficient participants were females, with *p* = 0.035 (Figure 2). 

### 3.5. Association of Household Food Security and Dietary Diversity with Vitamin B12 Intake and Serum Level

According to Table 4, more than 65% of the food-insecure participants had inadequate B12 intake and presented low serum levels of B12, with *p* = 0.000 and *p* = 0.06, respectively. Around 62.7% of the participants with inadequate B12 intake consumed two meals or fewer the day before the data collection, with *p* = 0.000, and reported their consumption patterns as fewer meals than usual, with *p* = 0.005. Moreover, 66.8% of participants with inadequate B12 intake had a low DD (*p* = 0.00) associated with low levels of serum B12 (*p* = 0.031). 

### 3.6. Dietary Sources of Vitamin B12 and Associations with FI

**Figure 3:**
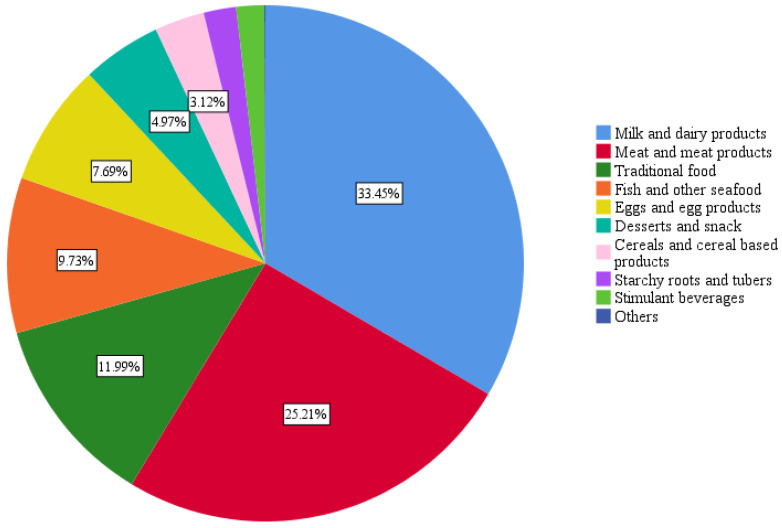
Contribution of the food groups to the daily vitamin B12 intake in the overall population.

**Figure 4:**
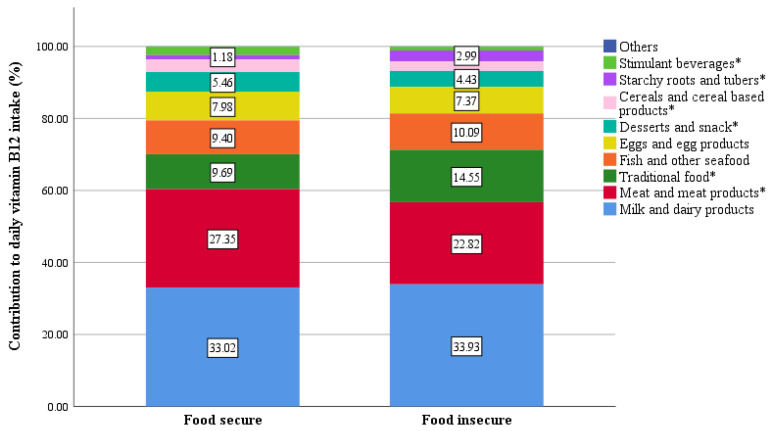
Contribution of the food groups to the daily vitamin B12 intake by household food security. * Food groups with a significant difference in contribution to B12 between the food-secure and the food-insecure.

The mean levels indicating the percentage of the contribution of the different food groups to the daily recommended B12 intake is presented in Figure 3. Milk and dairy products contributed the most to the study population’s daily B12 intake (33.45%), followed by meat and meat products (25.21%), and traditional food (11.99%). According to Figure 4, significantly greater contributions to the daily B12 intake were observed from meat and meat products in food-secure households (27.35%) compared to food-insecure households (22.82%), with *p* = 0.004. On the other hand, traditional food contributed significantly more vitamin B12 in food-insecure households (14.55%) than food-secure households (9.69 ± 9.3), with *p* = 0.000. The complete data for the B12 contributors is presented in Table S2.

### 3.7. Determinants of Inadequacy and Insufficient Serum Levels of B12 

A binary logistic regression analysis was performed to determine the factors that contributed to the inadequacy and insufficient serum levels of B12 (Table 5) in the study population. 

The variables that were significantly associated with the B12 intake, as determined from the bivariate analysis (*p* < 0.05), included the sex, marital status, household CI, number of children, education level, current occupation, household monthly income, disease status, food security status, number of meals consumed the day before, evaluation of number of meals reported, and DD of the participants. They were entered in the model (a backward stepwise method). The results of the bivariate analysis can be found in Table S3. Being female increased the likelihood of having inadequate intake in B12 levels by around threefold (OR = 2.845, 95% confidence interval (CI) = 1.757–4.606, and *p* = 0.000), and the unemployed had an 80.6% higher chance of having inadequate B12 intake (OR = 1.806, 95% CI = 1.129–2.884, and *p* = 0.014). Furthermore, being food-insecure increased the likelihood of inadequate intake of B12 by nearly twofold (OR = 2.145, 95% CI = 1.381–3.333, and *p* = 0.001), and the likelihood of inadequate intake of B12 was nearly threefold higher in participants with a low DD (OR = 3.034, 95% CI = 1.924–4.783, and *p* = 0.000).

The variables that were significantly associated with the serum levels of B12 from the bivariate analysis (*p* < 0.05) were residency, occupation, chicken, canned fish, DD, and B12 intake, and were entered in the model (the backward stepwise method) (Table S3). The likelihood of having low levels of serum B12 was nearly threefold higher in the participants residing in Beqaa (including Baalbek-Hermel) compared to those residing in Beirut (OR = 2.856, 95% CI = 1.018–8.017, and *p* = 0.046). Moreover, the participants with inadequate B12 intake were 78.8% more likely to have low serum levels of B12 compared to the participants with adequate intake (OR = 1.788, 95% CI = 1.206–2.650, and *p* = 0.004). Regarding our population’s consumption of foods of animal origin, the average consumption of eggs and egg products was higher than its pre-crisis supply (from 2010 to 2019) as reported by the FAOSTAT Food balances database [25] (Table 6). In contrast, there was a decrease in the consumption of milk and dairy products, meat and meat products, and fish and other seafood. These findings indicate a considerable decline in the consumption of most animal-based foods compared to the pre-crisis period.

### 3.8. Time Trends in Vitamin B12 Consumption and Serum Levels before and Amidst the Food Insecurity Situation

**Figure 5:**
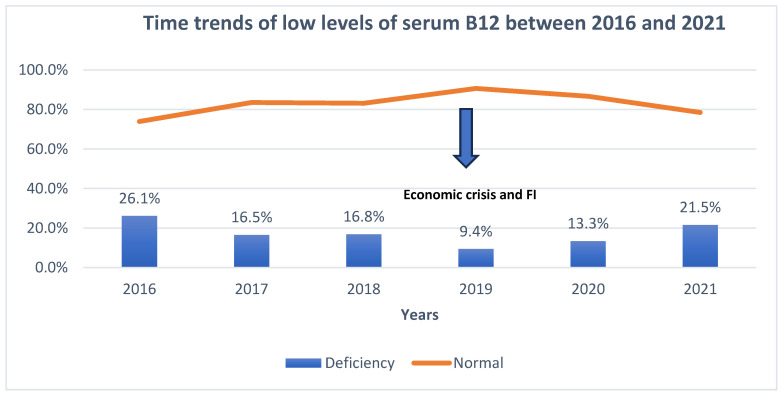
Time trends in sufficiency/insufficiency of the serum levels of B12 between 2016 and 2021. (Insufficient levels of serum B12 were defined as less than 300 pg/mL using the same assays).

Regarding the consumption of foods of animal origin, the average consumption of eggs and egg products was higher than its pre-crisis supply (2010 to 2019) as reported by the FAOSTAT Food balances database [25] (Table 6). In contrast, there was a decrease in the consumption of milk and dairy products, meat and meat products, and fish and other seafood. These findings indicate a considerable decline in the consumption of most animal-based foods compared to the pre-crisis period (Table 6). Similarly, compared to the period preceding the economic crisis time, a decline in the serum levels of B12 was observed accordingly since 2019, the time of economic crisis (Figure 5). 

## 4. Discussion

This study is the first of its kind to examine the prevalence of low levels of serum B12 and inadequate intake of B12 food sources in a nationally representative sample of Lebanese adults amid the ongoing crisis and to investigate how these people are impacted by FI. It also aimed to explore other predictors of low levels of serum B12 and its related low dietary intake. The results showed that over half of the participants had inadequate intake of B12 food sources and low levels of serum B12, with females being more affected than males. FI and a low DD were also prevalent in about half of the households. These findings show a deterioration in Lebanon’s FI situation, with an increase of 26.5% in FI prevalence compared to the previously reported WFP estimate between April and May 2020 [28] Moreover, inadequate B12 intake was more likely in participants who were female, unemployed, food-insecure, and had a low DD, while predictors of low serum levels of B12 included residing in Beqaa and having inadequate B12 dietary intake.

Additionally, more than half of the study participants (54.3%) reported consuming two meals or fewer on the day preceding the survey. This finding aligns with a recent study, which found that 55.3% of Lebanese households consumed fewer than two meals per day [6,7]. Furthermore, our study revealed a low DD in 56.3% of the households, representing an increase of over 3% compared to the results of a nationally representative study conducted in 2021 (53%) [6,7].

These shifts in dietary patterns are likely attributable to the sharp drop in the value of the Lebanese pound, coupled with the significant food price increases which has affected the affordability of these foods. For instance, according to the Lebanese Ministry of Economy, there has been a notable increase in the prices of meats and derivatives between September 2019 and 2020 (134%), between September 2020 and 2021 (195%), and between September 2021 and 2022 (148%) [30]. Similarly, the prices of eggs and dairy products increased within these three timeframes [30]. This situation is not unique to Lebanon, as individuals in numerous regions worldwide struggle to afford animal-based foods due to their high cost [30,31]. For instance, household FI among adults in Canada was associated with the consumption of lower-quality diets characterized by consistently lower nutrient intake and reduced consumption of milk products and sometimes meat and meat alternatives [32]. 

In this present study, the overall population’s mean for B12 intake was 2.9 μg/day, which is considerably lower than that of a previous Lebanese study conducted in 2009 on adults aged 20 y and over (4.6 μg/day) [33]. This is reflected in the previously discussed change in dietary patterns affected by the economic crisis. Regardless, our mean B12 intake is above the Recommended Dietary Allowance (RDA) of 2.4 μg/day for adults. Despite that, more than half of the participants (52.5%) had inadequate B12 intake, meaning they failed to meet the RDA. Regionally, this result was found to be higher than that of Iran (0.8%) [34] but lower than that of Saudi Arabia (78.7%) [35]. Internationally, our result was higher than that of most African countries for which data are available (<50%) [36], as well as Sri Lanka (41.2%) [37], Slovenia (31.7%) [38], Nepal (17.1%) [37], Pakistan (12%) [37], Ecuador (12%) [39], the Netherlands (≤6%) [40], the US (4%) [41], the UK (≤2%) [42], Spain (1%) [43], and Ireland (<1%) [44]. On the other hand, our result was lower than that of Bangladesh (73.5%) [45], India (60.3%) [46], and some African countries, including Ethiopia, Guinea-Bissau, Lesotho, and Liberia (>90%) as well as Malawi, Niger, Nigeria, Rwanda, Tanzania, and Zimbabwe (>60%) [47]. These variations are unsurprising since the US, United Kingdom (UK), and European nations are developed countries where meat, an important source of B12, often serves as the primary ingredient in numerous meals, [47] contrary to developing countries like Bangladesh or certain African countries that suffer from FI, hunger, and malnutrition [47]. 

Regarding the food group contributions to daily B12 intake, milk and dairy products contributed the most to our population’s daily B12 intake (33.45%), followed by meat and meat products (25.21%) and traditional food (11.99%). This was similar to findings from national nutrition surveys in the UK [42], the Netherlands [40] and Australia [48], which reported the highest contribution from milk and dairy products (30–38%) followed by meat and meat products (27–30%). On the other hand, Denmark [49], Ireland [44], and Slovenia [38] reported meat and meat products (35–44.5%) as contributing the most to B12 intake followed by milk and dairy products (19.5–29%), while a study in Spain found that meat (55.8%) contributed the most to B12 intake followed by fish (23.8%) [50]. When comparing contributions between food-secure and food-insecure households, a significantly higher contribution of meat and meat products to daily B12 intake was observed in food-secure households (27.35%) compared to food-insecure households (22.82%). Conversely, traditional food contributed significantly more to daily B12 intake in food-insecure households (14.55%) than in food-secure households (9.69%). This could be explained by the fact that meat and meat products are more expensive and possibly harder to access by food-insecure households, whereas traditional Lebanese foods, which comprise yogurt or minced meat (depending on the type of dish), are probably more sought out by food i-secure households as a cost-effective alternative. 

When assessing the serum levels of B12 in our population, the overall mean serum level of B12 was 297.3 pg/mL, which falls at the higher end of the insufficiency category. Furthermore, 61.1% presented with a low level (23%) or an intermediate level (38.1%). When compared to other studies, regionally, the prevalence of low levels appeared to be higher in Jordan (30.1%) [51] and Iran (27.1%) [34] but lower in Saudi Arabia (7.5%) [52] than our study finding (23%). Internationally, all studies except India (34%) [46] reported a lower prevalence of low serum levels of B12, including Bangladesh (~<15%) [53], Liechtenstein (13.4%) [54], Panama (11.8%) [55], Spain (10.9%) [43], Costa Rica (11.2%) [56], the UK (6%) [42], Ireland (6%) [57], Canada (~<6%) [58], Ecuador (5.46%) [39], the US (~3–4% in 1999–2002 [41] and ~<7% in 2003–2006 [59]), France (3.3%) [60], Australia (2.3%) [61], and Korea (~<2%) [62]. As for our alarming prevalence of low to intermediate levels of B12 combined together (61.1%), it unexpectedly appeared to be the highest of all published studies that assessed the prevalence of low serum levels of B12 to our knowledge, including Jordan (43.5%) regionally [52]; and Costa Rica (42.4%) [56], Panama (37.5%) [55], Canada (~<28%) [58], the US (17–20%) [41], Australia (14.7%) [61], Brazil (6.4%) [63], and Finland (4.7%) [64] internationally. This raises public health concerns regarding the nutritional status of the Lebanese population. However, the collective evidence indicates that low serum levels of B12 are a significant concern in various populations worldwide, including Lebanon. Seeing as our study, unlike other studies, adopted relatively rigorous exclusion criteria for medical conditions and medications known to potentially impact B12 absorption, it is highly unlikely that the remarkably high prevalence of low serum levels of B12 in our study is attributed to malabsorption; rather, the low serum levels of B12 are more likely related to inadequate B12 intake, which was confirmed in the multivariate analysis as a predictor of the low serum levels of B12. This highlights the severity of the situation in Lebanon, which left people prone to nutritional inadequacies and deficiencies.

Regarding the sex differences in consumption, females consumed significantly less than males from most food groups, markedly those of animal origin. Females were three times more likely to have B12 inadequate intake compared to males who generally have higher requirements than females [65]; however, the observed difference coming particularly from foods of animal origin supports the proposition that females purposely decrease their consumption of these foods in favor of males or younger individuals within the same household [66]. A study in Lebanon has previously discussed this link between household FI and compromised diet quality among Lebanese mothers [67], emphasizing that household food security does not ensure food security for all household members [68]. Similar findings in Pakistan suggest that biased food allocation places women at a higher risk of micronutrient deficiencies, even in food-secure households [69]. Interestingly, females had a significantly lower mean B12 intake than males (2.3 μg/day vs. 3.8 μg/day), and the majority of participants with inadequate intake (71.7%) were females. Our finding was consistent with numerous studies that reported lower B12 intake among females compared to males [37,43,50,70,71,72]. Furthermore, the most participants with low serum levels of B12 in the current study were females (68.6%), which was expected given the lower dietary B12 intake of females compared to males. The results from some studies aligned with ours [52,53,54], while others found a higher prevalence of low serum levels of B12 in males [73]. In short, females in Lebanon were found to be susceptible to low dietary intake and low serum levels of B12 in our population, presumably due to altered dietary patterns. Since compromised maternal serum levels of B12 before pregnancy has been shown to impact child development during pregnancy and infancy [74], it is important to improve B12 serum levels in females to prevent a deficiency in future generations of Lebanese adults. 

Additionally, food-insecure individuals and those with a low DD were two and three times more likely to have inadequate dietary intake of B12, respectively. The DD refers to the variety of different foods consumed within a diet over a given period of time, and uncertain access to food may lead to a less varied and nutritious diet potentially lacking in B12-rich foods, leading to low dietary intake. While no studies in the literature discussed the association between FI and B12 intake using multivariate analysis, few reported results using bivariate analysis. Contrary to our result, in one US study, there was apparently no significant difference in the prevalence of inadequate B12 intake by food security status in adults (*p >* 0.05) [16], while another study conducted on a rural population in the US found a significant difference in the prevalence of adequate B12 intake between food-insecure and food-secure adults (*p* = 0.019) [17]. Likewise, the prevalence of inadequate B12 intake in a Canadian population was considerably higher for those in food-insecure than food-secure households in most age/sex groups, although no *p*-value was provided [32]. Furthermore, the unemployed had an 80.6% higher chance of having inadequate B12 intake compared to those who were employed, possibly because limited financial resources may restrict access to a diverse range of nutrient sources, including vitamin B12. Similar to our result, one study found that those who were employed had a lower risk for inadequate daily B12 intake compared to retired participants, with the association being marginally significant [37].

The individuals residing in Beqaa were three times more likely to have low levels of serum B12 compared to those residing in Beirut. Findings on regional differences in the prevalence of low levels of B12 were noted by other studies. For instance, one study in Jordan found those living in the north more likely to have low levels of B12 than those living in the middle or south regions [51], and another found the prevalence of low serum levels of B12 was three- to fivefold higher in the north than in the south of China (*p* < 0.001) [75]. Moreover, participants with inadequate B12 intake were 78.8% more likely to have low serum levels of B12 compared to those with adequate intake [75]. The results from other studies were conflicting, where some aligned with our results [43,49,76] whereas others found no relation between B12 intake and its related serum levels [77,78,79]. This may be related to the multifactorial causes of low serum levels of B12—including genetic, medical, and dietary factors—playing varying roles across countries, with the dietary factor being the main player in our study given the Lebanese FI context. 

In this present study, FI did not emerge as a significant predictor of low levels of serum B12. However, it is worth noting that participants from food-insecure households exhibited a higher prevalence of low serum levels of B12 (65.7%) compared to their food-secure counterparts (56.8%), although the association did not reach statistical significance (*p* = 0.064) but approached a level of potential significance (*p* = 0.05). This result was similar to that of a US study which showed no significant difference between the means levels of serum B12 among adults from food-insecure and food-secure families (*p* = 0.84) [16]. In our study population, since FI was identified as a significant predictor of inadequate B12 intake, which in turn was found to be a significant predictor of low serum levels of B12, it can be inferred that FI has an indirect effect on low levels of B12. This highlights the importance of addressing the FI situation in Lebanon to mitigate its impact on B12 intake and, in turn, serum levels in the Lebanese population, especially in high-risk groups observed in our study like females, the unemployed, and Beqaa residents.

Based on these insights, interventions aimed at reducing the risk of low serum levels of B12 among Lebanese adults may involve the targeted supplementation of vitamin B12 in high-risk groups and fortification programs for specific food items with vitamin B12. Published guidelines on food [80] and flour fortification [81] with micronutrients could facilitate such a program. Additionally, food assistance programs, including in-kind food aid or food vouchers, can help address FI. Furthermore, it is crucial to enhance public awareness regarding the symptoms of low serum levels of B12 and emphasize the significance of preventive screening campaigns, particularly targeting high-risk groups to mitigate the long-term negative health outcomes and economic burdens associated with low serum levels of B12. Moving forward, longitudinal research is imperative to gain insights into the long-term nature of FI and its implications for nutritional well-being in the Lebanese population, given the worsening economic crisis. Moreover, future studies should consider utilizing additional biomarkers of low serum levels of B12, such as methylmalonic acid or homocysteine, to obtain more accurate deficiency estimates [8]. In addition, due to the scarcity of the literature in this area, more studies globally and regionally should investigate the impact of FI on B12 serum levels in their respective populations to provide a more comprehensive understanding of the global situation regarding this issue.

One of the major strengths of this study is that it was the first to assess the prevalence of low serum levels of B12 in a nationally representative sample of Lebanese adults filling a significant gap in the existing literature. This study also stands out as the first of its kind to examine the impact of FI in Lebanon on a micronutrient deficiency among the adult population following the economic crisis, providing valuable insights into the consequences of this pressing issue. Additionally, strict exclusion criteria were adopted, minimizing confounding effects and strengthening the internal validity of our findings. Despite these strengths, our study is not without limitations. Our study primarily relied on self-reported data which is subject to recall bias and potential inaccuracies, especially under- and over-reporting of food consumption. Efforts were made to mitigate these limitations, such as using trained interviewers and asking straightforward questions. 

## 5. Conclusions

Our research highlights the alarming prevalence of low serum levels of B12 among Lebanese adults. The higher prevalence of low serum levels of B12 in Lebanon compared to most countries indicates that it is a pressing public health concern in the nation. Interestingly, we observed that individuals experiencing FI were more likely to have a low dietary intake of B12-rich foods and were more likely to have low serum levels of B12. These findings suggest an indirect relationship between FI and low serum levels of B12 mediated by a low dietary intake of B12-rich foods. To address these concerns, interventions should encompass targeted supplementation, fortification programs, food assistance, and awareness and screening campaigns.

## Figures and Tables

**Table 1:** Sociodemographic characteristics of the overall study population and by sex.

Variable		Overall (n = 444)		Male (n = 183)		Female (n = 261)		
		Mean	SD	Mean	SD	Mean	SD	*p*-Value
**Age (y)**		34.1	12.7	33.8	12.2	34.4	13.1	0.649
		**N**	**%**	**N**	**%**	**N**	**%**	***p*-Value**
Age categories ^a^	18–24 y	149	33.6	55	36.9	94	63.1	0.005 *
	25–39 y	151	34.0	78	51.7	73	48.3	
	40–64 y	144	32.4	50	34.7	94	65.3	
	Total	444		183	41.2	261	58.8	
Residency	Akkar	31	7.0	11	35.5	20	64.5	0.006 *
	Mount Lebanon	179	40.3	73	40.8	106	59.2	
	Beqaa	27	6.1	5	18.5	22	81.5	
	North Lebanon	60	13.5	33	55.0	27	45.0	
	Baalbek-Hermel	20	4.5	10	50.0	10	50.0	
	South Lebanon	55	12.4	30	54.5	25	45.5	
	Beirut	32	7.2	10	31.3	22	68.8	
	Nabatieh	40	9.0	11	27.5	29	72.5	
Marital status	Single	202	45.5	81	40.1	121	59.9	0.128
	Married	223	50.2	97	43.5	126	56.5	
	Widowed	7	1.6	0	0.0	7	100.0	
	Divorced	12	2.7	5	41.7	7	58.3	
Household crowding index	No crowding	165	37.2	79	47.9	86	52.1	0.036 *
	Crowding	279	62.8	104	37.3	175	62.7	
Number of children	None	217	48.9	87	40.1	130	59.9	0.124
	1–3	159	35.8	74	46.5	85	53.5	
	More than 3	68	15.3	22	32.4	46	67.6	
Education level	Illiterate	3	0.7	1	33.3	2	66.7	1.000
	School	175	39.4	72	41.1	103	58.9	
	University	266	59.9	110	41.4	156	58.6	
Current occupation	Unemployed	218	49.1	40	18.3	178	81.7	<0.001 *
	Employed	226	50.9	143	63.3	83	36.7	
Monthly salary change since the start of economic crisis	No impact	129	29.1	63	48.8	66	51.2	<0.001 *
	Decline in salary	123	27.7	51	41.5	72	58.5	
	Increase in salary	70	15.8	42	60.0	28	40.0	
	Already have no salary	122	27.5	27	22.1	95	77.9	
Household monthly income	None	39	8.8	17	43.6	22	56.4	<0.001 *
	<LBP 1.5 million	58	13.1	15	25.9	43	74.1	
	≥LBP 1.5 million	211	47.5	79	37.4	132	62.6	
	≤USD 300	92	20.7	41	44.6	51	55.4	
	>USD 300	44	9.9	31	70.5	13	29.5	

* Significant at *p*-value < 0.05. ^a^ Classification realized in such a way to ensure an approximately equal distribution across categories.

**Table 2:** Health characteristics of the overall study population and by sex.

Variable		Overall (n = 444)		Male (n = 183)		Female (n = 261)		
		Mean	SD	Mean	SD	Mean	SD	*p*-Value
Weight (kg)		73.8	17.1	81.8	16.6	68.3	15.1	<0.001 *
Height (cm)		165.3	9.4	173.5	7.0	159.5	5.9	<0.001 *
Body mass index (kg/m^2^)		27.0	5.8	27.2	5.3	26.9	6.1	0.626
		**N**	**%**	**N**	**%**	**N**	**%**	***p*-Value**
BMI classification	Underweight	19	4.3	4	21.1	15	78.9	0.084
	Normal	150	33.8	57	38.0	93	62.0	
	Overweight and Obese	275	61.9	122	44.4	153	55.6	
Disease status	No disease	333	75.0	150	45.0	183	55.0	0.005 *
	Having disease	111	25.0	33	29.7	78	70.3	
	Cardiovascular disease	14	12.6	5	15.2	9	11.5	
	Diabetes	3	2.7	1	3.0	2	2.6	
	Hypertension	34	30.6	16	48.5	18	23.1	
	Kidney disease	4	3.6	2	6.1	2	2.6	
	Liver disease	1	0.9	1	3.0	0	0.0	0.004
	Osteoporosis	14	12.6	1	3.0	13	16.7	
	Asthma/Respiratory diseases	12	10.8	2	6.1	10	12.8	
	Anemia	36	32.4	5	15.2	31	39.7	
	Others ^a^	32	28.8	6	18.2	26	33.3	
History of COVID-19 infection	No	126	28.4	52	41.3	74	58.7	0.296
	Yes	238	53.6	104	43.7	134	56.3	
	Don’t know	80	18.0	27	33.8	53	66.3	
COVID-19 vaccination	No	142	32.0	51	35.9	91	64.1	0.123
	Yes	302	68.0	132	43.7	170	56.3	

* Significant at *p*-value < 0.05. ^a^ Includes other self-reported diseases: (1) allergies (seasonal, food, dust, skin); (2) vertebral column problems; (3) sarcoidosis; (4) migraines; (5) thyroid disease; (6) gastrointestinal problems; (7) psychological conditions; (8) neurological conditions; (9) hypovitaminosis D; (10) hypocalcemia; (11) iron deficiency; (12) urinary tract infection; (13) hypercholesterolemia; (14) Raynaud’s syndrome; (15) varicose veins; (16) autoimmune diseases; (17) cancer; (18) thrombosis; (19) polycystic ovarian syndrome.

**Table 3:** Assessment of household food security and dietary diversity in the overall population and by sex.

Variable		Overall (n = 444)		Male (n = 183)		Female (n = 261)		
		N	%	N	%	N	%	*p*-Value
Food security status	Food-secure	234	52.7	102	55.7	132	50.6	0.28
	Food-insecure	210	47.3	81	44.3	129	49.4	
Number of meals consumed the day before	2 meals or fewer	241	54.3	100	41.5	141	58.5	0.923
	3 meals or more	203	45.7	83	40.9	120	59.1	
Evaluation of number of meals reported	Fewer than usual	107	24.1	39	36.4	68	63.6	0.491
	As usual	330	74.3	141	42.7	189	57.3	
	More than usual	7	1.6	3	42.9	4	57.1	
Dietary diversity	Low	250	56.3	102	40.8	148	59.2	0.846
	High	194	43.7	81	41.8	113	58.2	

**Table 4:** Association of household food security and dietary diversity with vitamin B12 intake and serum levels.

Variable		Vitamin B12 Intake **					B12 Serum Levels ***				
		Adequate		Inadequate			Normal		Insufficient		
		N	%	N	%	*p*-Value	N	%	N	%	*p*-Value
Food security status	Food-secure	140	59.8	94	40.2	0.000 *	101	43.2	133	56.8	0.064
	Food-insecure	71	33.8	139	66.2		72	34.3	138	65.7	
Number of meals consumed the day before	2 meals or fewer	90	37.3	151	62.7	0.000 *	89	36.9	152	63.1	0.379
	3 meals or more	121	59.6	82	40.4		84	41.4	119	58.6	
Evaluation of number of meals reported	Fewer than usual	37	34.6	70	65.4	0.005 *	35	32.7	72	67.3	0.291
	As usual	171	51.8	159	48.2		135	40.9	195	59.1	
	More than usual	3	42.9	4	57.1		3	42.9	4	57.1	
Dietary diversity	Low	83	33.2	167	66.8	0.000 *	86	34.4	164	65.6	0.031 *
	High	128	66.0	66	34.0		87	44.8	107	55.2	

* Significant at a *p*-value < 0.05. ** B12 intake was categorized according to the RDA cutoff as adequate (≥2.4 μg/d) and inadequate (<2.4 μg/d). *** The serum B12 levels were categorized as normal (>300 pg/mL or 221.4 pmol/L), intermediate (200–300 pg/mL or 147.6–221.4 pmol/L), and low serum levels (<200 pg/mL or <147.6 pmol/L). Insufficient indicates an intermediate to a low serum level (<300 pg/mL or 221.4 pmol/L).

**Table 5:** Determinants of inadequate vitamin B12 intake (<2.4 μg/d) and insufficient levels of serum B12 (<300 pg/mL or 221.4 pmol/L).

Determinants of B12 Intake (Adequate (Reference) vs. Inadequate)		OR	95% CI		*p*-Value
			Lower	Upper	
Sex	Male (Reference)				
	Female	2.845	1.757	4.606	<0.001 *
Current occupation	Employed (Reference)				
	Unemployed	1.806	1.129	2.884	0.014 *
Food security status	Food-secure (Reference)				
	Food-insecure	2.145	1.381	3.333	0.001 *
Number of meals consumed the day before	3 meals or more (Reference)				
	2 meals or fewer	1.497	0.951	2.358	0.081
Dietary diversity	High dietary diversity (Reference)				
	Low dietary diversity	3.034	1.924	4.783	<0.001 *
Determinants of Low Serum B12 Levels (Normal (Reference) vs. Insufficient)					
	Beirut (Reference)				
	North Lebanon	1.305	0.558	3.049	0.539
Residency	Mount Lebanon	0.817	0.374	1.788	0.614
	Beqaa	2.856	1.018	8.017	0.046 *
	South Lebanon	0.736	0.321	1.689	0.470
Vitamin B12 intake	Adequate (Reference)				
	Inadequate	1.788	1.206	2.650	0.004 *

* Significant at a *p*-value < 0.05.

**Table 6:** Comparison between food group consumption/supply pre-crisis, during 2020, and in the current study.

Food Groups	Food Supply Quantity (g/Capita/Day)		Food Consumption (g/Day)
	2010–2019 (Pre-Crisis)	2020	2022 (Current Study)
Eggs	8.8	12.46575	22.2
Fish and seafood	25.05753	23.89041	11.4
Meat	89.87945	80.79452	69.5
Milk and dairy products	200.1397	169.8082	184.5

## Data Availability

The data presented in this study are available upon request from the corresponding author.

## Supplementary Materials

The following supporting information can be downloaded at: https://www.mdpi.com/article/10.3390/nu16020226/s1. Table S1: Dietary intake of food groups obtained from the food frequency questionnaire and the mean of 2 non-consecutive 24 h recall in the overall population, by sex, and by age groups. Table S2: Percentage of contribution of food groups to daily vitamin B12 intake in the overall population and by household FI. Table S3: Associations of demographic and socioeconomic characteristics of the study population with vitamin B12 intake and status.
