# Supplementary material for: Prevalence and Time Trends of Low Serum B12 Levels and Inadequate B12 Dietary Intake in Lebanese Adults amidst the Food Insecurity Situation: Findings from a Nationally Representative Cross-Sectional Study
Source: Nutrients. 2024 Jan 10;16(2):226. doi: 10.3390/nu16020226 (PMC10818865; doi:10.3390/nu16020226)
Supplement: Supplementary file 1 [file nutrients-16-00226-s001.zip › nutrients-2790518-supplementary.pdf]

Table S1. Dietary intake of food groups obtained from the food frequency questionnaire and the mean of 2 non-consecutive 24h recall in the overall population, by sex, and by age groups.

Food groups	Dietary intake (g/day)							Dietary intake (g/day)						
	Overall (n=444)		Male (n=183)		Female (n=261)			18-24 y (n=149)		25-39 y (n=151)		40-64 y (n=144)		
	Mean	SD	Mean	SD	Mean	SD	*p-value*	Mean	SD	Mean	SD	Mean	SD	*p-value*
**Cereals and cereal based products**	317.2	134.9	390.6	140.0	265.7	104.0	0.000*	338.8	142.9	307.5	130.5	304.9	128.9	0.055
**Fruit and fruit products**	254.3	285.0	275.9	286.7	239.2	283.3	0.182	255.8	272.9	215.7	210.2	293.4	353.6	0.064
**Vegetables and vegetable products**	206.5	117.8	197.8	115.4	212.6	119.3	0.191	217.4	120.2	189.2	118.5	213.3	113.1	0.081
**Milk and dairy products**	184.5	164.7	208.3	186.1	167.8	145.9	0.011*	190.3	180.4	191.1	159.6	171.6	153.0	0.521
**Meat and meat products**	69.5	75.1	94.1	98.5	52.2	45.8	0.000*	74.6	57.3	77.3	82.2	56.0	81.9	0.030*
**Legumes and pulses**	66.9	54.8	68.2	62.2	65.9	49.0	0.675	64.8	61.0	60.5	46.6	75.7	55.2	0.049*
**Desserts and snack**	65.4	114.1	77.7	159.2	56.7	65.2	0.092	100.1	176.0	59.2	61.1	35.9	46.4	<0.001*
**Starchy roots and tubers**	50.5	43.5	54.3	42.4	47.8	44.1	0.118	55.2	49.0	48.8	38.4	47.3	42.3	0.248
**Herbs, spices and condiments**	47.9	44.1	50.1	46.1	46.3	42.7	0.373	48.4	46.6	45.7	41.6	49.6	44.2	0.735
**Traditional food**	33.6	35.5	38.3	39.4	30.2	32.2	0.018*	40.6	45.7	30.5	29.2	29.5	27.8	0.012*
**Eggs and egg products**	22.2	32.6	32.4	44.3	15.1	17.8	0.000*	26.3	32.6	20.5	33.4	19.8	31.5	0.165
**Sugar and confectionery**	19.0	22.2	20.5	23.8	17.9	20.9	0.235	21.0	24.8	17.6	19.8	18.3	21.6	0.365
**Fat and oils of animals and vegetables**	11.5	11.7	12.2	13.8	11.0	10.0	0.289	10.6	10.1	10.1	9.4	14.0	14.6	0.009*
**Fish and other seafood**	11.4	21.0	16.9	28.2	7.6	12.6	0.000*	13.1	26.9	12.1	18.7	8.9	15.6	0.206
**Nuts and oilseeds**	5.0	12.5	6.5	13.1	4.0	11.9	0.043*	6.5	18.1	4.3	8.6	4.2	7.8	0.213
**Drinking water**[a]	1440.5	843.2	1803.3	865.8	1186.1	727.3	0.000*	1318.8	746.5	1470.7	770.0	1534.8	989.2	0.078
**Stimulant beverages**[a]	546.8	427.4	543.6	454.4	549.1	408.2	0.893	514.2	387.0	548.3	435.1	579.0	458.3	0.431
**Non-Alcoholic beverages**[a]	70.6	120.9	97.0	160.6	52.1	77.7	0.001*	86.1	143.7	75.3	120.8	49.7	89.1	0.031*
**Fruit and vegetable juices** [a]	66.3	96.1	77.7	108.9	58.2	85.3	0.035*	93.2	123.5	67.1	88.5	37.5	54.8	<0.001*
**Alcoholic beverages**[a]	0.6	5.9	1.5	9.1	0.0	0.0	0.031*	0.1	0.6	1.1	8.3	0.6	5.8	0.283

* Significant at p-value < 0.05

[a] Beverages are presented in mL/day

Table S2. Percentage of contribution of food groups to daily vitamin B12 intake in the overall population and by household FI.

Food groups[a]	Contribution to daily vitamin B12 intake (%)						
	Overall		Food secure		Food insecure		
	Mean	SD	Mean	SD	Mean	SD	*p-value*
**Milk and dairy products**	33.45	19.8	33.02	18.4	33.93	21.3	0.633
**Meat and meat products**	25.21	16.5	27.35	15.9	22.82	16.8	0.004*
**Traditional food**	11.99	13.9	9.69	9.3	14.55	17.3	<0.001*
**Fish and other seafood**	9.73	11.5	9.40	9.8	10.09	13.2	0.535
**Eggs and egg products**	7.69	8.2	7.98	8.2	7.37	8.3	0.442
**Desserts and snack**	4.97	5.3	5.46	4.8	4.43	5.8	0.041*
**Cereals and cereal based products**	3.12	4.0	3.50	4.0	2.69	4.0	0.033*
**Starchy roots and tubers**	2.04	5.8	1.18	2.6	2.99	7.8	0.002*
**Stimulant beverages**	1.74	6.3	2.35	7.8	1.06	3.9	0.026*
**Other[b]**	0.07	0.1	0.07	0.1	0.07	0.1	0.878

* Significant at p-value < 0.05

[a] Food groups with no contribution to vitamin B12 intake at all were not included in the table

[b] Includes the following food groups with respective negligible contributions: Herbs, spices and condiments (0.0618%), fat and oils of animals and vegetables (0.0071%), and alcoholic beverages (0.0019%)

Table S3. Associations of demographic and socioeconomic characteristics of the study population with vitamin B12 intake and status.

Variable		Vitamin B12 intake					Vitamin B12 status				
		Adequate		Inadequate			Normal		Deficient		
		N	%	N	%	*p-value*	N	%	N	%	*p-value*
**Sex**	Male	117	63.9	66	36.1	0.000*	71	38.8	112	61.2	1.000
	Female	94	36.0	167	64.0		102	39.1	159	60.9	
**Age categories**	18-24 y	80	53.7	69	46.3	0.092	53	35.6	96	64.4	0.544
	25-39 y	72	47.7	79	52.3		60	39.7	91	60.3	
	40-64 y	59	41.0	85	59.0		60	41.7	84	58.3	
**Residency**	Mount Lebanon	91	50.8	88	49.2	0.235	79	44.1	100	55.9	0.007*
	Beirut	12	37.5	20	62.5		12	37.5	20	62.5	
	South Lebanon	46	48.4	49	51.6		44	46.3	51	53.7	
	North Lebanon	36	39.6	55	60.4		29	31.9	62	68.1	
	Beqaa	26	55.3	21	44.7		9	19.1	38	80.9	
**Marital status**	Single	109	54.0	93	46.0	0.017*	75	37.1	127	62.9	0.782
	Married	98	43.9	125	56.1		89	39.9	134	60.1	
	Widowed	1	14.3	6	85.7		3	42.9	4	57.1	
	Divorced	3	25.0	9	75.0		6	50.0	6	50.0	
**Household crowding index**	No crowding	94	57.0	71	43.0	0.002*	72	43.6	93	56.4	0.131
	Crowding	117	41.9	162	58.1		101	36.2	178	63.8	
**Number of children**	None	116	53.5	101	46.5	0.009*	81	37.3	136	62.7	0.588
	1-3	73	45.9	86	54.1		67	42.1	92	57.9	
	More than 3	22	32.4	46	67.6		25	36.8	43	63.2	
**Education level**	Illiterate	1	33.3	2	66.7	0.000*	0	0.0	3	100.0	0.468
	School	61	34.9	114	65.1		67	38.3	108	61.7	
	University	149	56.0	117	44.0		106	39.8	160	60.2	
	Unemployed	72	33.0	146	67.0	0.000*	73	33.5	145	66.5	0.025*

**Current occupation**	Employed	139	61.5	87	38.5		100	44.2	126	55.8	
**Monthly salary change after economic crisis**	Increase or already have no salary	91	47.4	101	52.6	1.000	73	38.0	119	62.0	0.768
	Decline or no change	120	47.6	132	52.4		100	39.7	152	60.3	
**Household monthly income**	None	14	35.9	25	64.1	0.000*	15	38.5	24	61.5	0.121
	< 1.5 million L.B.P.	14	24.1	44	75.9		14	24.1	44	75.9	
	≥1.5 million L.B.P.	99	46.9	112	53.1		84	39.8	127	60.2	
	≤300 USD	54	58.7	38	41.3		39	42.4	53	57.6	
	>300 USD	30	68.2	14	31.8		21	47.7	23	52.3	

* Significant at p-value < 0.05
